# Association between an 8q24 locus and the risk of colorectal cancer in Japanese

**DOI:** 10.1186/1471-2407-9-379

**Published:** 2009-10-26

**Authors:** Keitaro Matsuo, Takeshi Suzuki, Hidemi Ito, Satoyo Hosono, Takakazu Kawase, Miki Watanabe, Kohei Shitara, Koji Komori, Yukihide Kanemitsu, Takashi Hirai, Yasushi Yatabe, Hideo Tanaka, Kazuo Tajima

**Affiliations:** 1 Division of Epidemiology and Prevention, Aichi Cancer Center Research Institute, Nagoya, Japan; 2 Director, Aichi Cancer Center Research Institute, Nagoya, Japan; 3 Department of Medical Oncology, Aichi Cancer Center Hospital, Nagoya, Japan; 4 Department of Gastroenterological Surgery, Aichi Cancer Center Hospital, Nagoya, Japan; 5 Department of Pathology and Molecular Diagnosis, Aichi Cancer Center Hospital, Nagoya, Japan; 6 Department of Epidemiology, Nagoya University Graduate School of Medicine, Nagoya, Japan; 7 Department of Medical Oncology and Immunology, Nagoya City University Graduate School of Medical Science, Nagoya, Japan

## Abstract

**Background:**

A genome-wide association study (GWAS), which assessed multiple ethnicities, reported an association between single nucleotide polymorphisms in the 8q24 region and colorectal cancer risk. Although the association with the identified loci was strong, information on its impact in combination with lifestyle factors is limited.

**Methods:**

We conducted a case-control study in 481 patients with colorectal cancer (CRC) and 962 sex-age matched non-cancer controls. Data on lifestyle factors, including diet, were obtained by self-administered questionnaire. Two 8q24 loci, rs6983267 and rs10090154, were assessed by the TaqMan method. Associations were then assessed by multivariate logistic regression models that considered potential confounders.

**Results:**

We found an increased risk of CRC with rs6983267 but not with rs10090154. An allelic OR was 1.22 (1.04-1.44, p for trend = 0.014), which remained significant after adjustment for confounders (OR = 1.25). No statistically significant interaction with potential confounding factors was observed.

**Conclusion:**

The polymorphism rs6983267 showed a significant association with CRC in a Japanese population. Further investigation of the biological mechanism of this association is warranted.

## Background

Colorectal cancer (CRC) remains major cancer worldwide [1]. Although numerous epidemiological and biological studies have revealed risk/protective factors for CRC, present knowledge is still insufficient to allow the disease to be overcome, and the struggle to elucidate mechanisms is ongoing.

Recently, several a number of genome-wide association studies (GWAS) have revealed an association between variants on chromosome 8q24 and several sites of cancer, including CRC [2-11]. Each study showed that rs6983267 resides in 128.47-128.54 MB on Chromosome 8, denoted as 'region 3,' [7] and consistently associated with CRC [6,9,12]. This association was confirmed in a subsequent large-scale replication study in Caucasians [13-18]. Most of these CRC GWASs were conducted in Caucasian populations, however, and the data available for Asian populations is limited especially about possible gene-environment interaction [6,19].

The aim of the present case-control study was to clarify the impact of rs6983267 on CRC risk in a Japanese population. In addition, we explored the gene-environmental interaction between potential confounders and rs6983267.

## Methods

### Subjects

Cases were 481 patients who were histologically diagnosed with CRC (245 with colon cancer, 231 with rectum cancer) between January 2001 and November 2005 at Aichi Cancer Center Hospital (ACCH) and who had no prior history of cancer. Controls were first-visit outpatients at ACCH during the same periods who were confirmed to have no cancer or a prior history of neoplasm. Controls were randomly selected and matched for sex and age (± 4 years) with a 1:2 case-control ratio (*n *= 962). The subjects were selected from the database of the Hospital-based Epidemiologic Research Program at Aichi Cancer Center (HERPACC). The framework of HERPACC has been described elsewhere [20,21]. Briefly, all outpatients aged 20-79 years were asked at first visit to fill out a questionnaire regarding their lifestyle and provided 7 ml of blood. A trained interviewer checked the completion of each questionnaire. Approximately 95% of eligible subjects completed the questionnaire and 55% provided blood samples. Some 30% of first-visit outpatients were diagnosed at ACCH as having cancer. Under the assumption that the non-cancer population within HERPACC will visit ACCH if they develop cancer in the future, we defined non-cancer first-visit outpatients as those from among whom such cases may arise. Our previous study confirmed that the lifestyle patterns of first-visit outpatients matched the profile of a group randomly selected from the general population of Nagoya City, conferring external validity on the study [22]. Written informed consent was obtained from all subjects and the ethics committee of ACC approved the study.

### Determination of the 8q24 loci genotype

DNA of each subject was extracted from the buffy coat fraction with a Blood Mini Kit (Qiagen K.K., Tokyo, Japan) and assessed using the polymerase chain reaction (PCR) TaqMan method [23] with the 7500 Fast Real-time PCR system (Applied Biosystems, Foster City, CA, USA). The probes used were specifically designed for rs6983267 and rs10090154 in 8q24. rs10090154 in the 8q24 'region 1' [7] was chosen because it showed a significant association for a Japanese population in Hawaii [6]. The quality of genotyping was assessed by duplicate analysis of 5% of random samples, with an agreement rate of 100%.

### Exposure data

Cumulative smoking dose was evaluated as pack-years, the product of the number of packs consumed per day and years of smoking. Smoking habit was classified into the three categories of never, pack-years < 20 (low-moderate) and ≥ 20 pack years (heavy). Consumption of types of alcoholic beverages (Japanese *sake*, beer, *shochu*, whiskey and wine) per occasion was determined with reference to the average number of drinks per day, which was then converted into a Japanese sake (rice wine) equivalent (one unit sake = 23 g ethanol) [24]. Daily ethanol consumption was estimated as the product of the frequency of alcohol beverage and average ethanol consumption occasion, and drinking habit was classified into the four categories of non-drinker, low (< 5 g/day), moderate (< 23 g/day) and heavy (≥ 23 g/day). Consumption of folate was determined using a semi-quantitative food frequency questionnaire (SQFFQ) as described in detail elsewhere [25]. Briefly, the SQFFQ consisted of 47 single food items with frequencies in the eight categories of never or seldom, 1-3 times/month, 1-2 times/week, 3-4 times/week, 5-6 times/week, once/day, twice/day, and 3+ times/day. Average daily intake of nutrients was estimated by multiplying the food intake (in grams) or serving size by the nutrient content per 100 grams of food as listed in the Standard Tables of Food Composition in Japan, 5^th ^edition. Consumption of supplemental folate was not considered in total consumption because the questionnaire for multi-vitamins was not quantitative. Energy-adjusted intake of nutrients was calculated by the residual method [26]. The SQFFQ was validated by reference to a 3-day weighted dietary record as a standard, which showed the reproducibility and validity to be acceptable [27,28]. The de-attenuated correlation coefficients for energy-adjusted intakes of folate were 0.36 in men and 0.38 in women. Body mass index (BMI) was calculated as the self-reported weight (kilograms) divided by the square of self-reported height (meters). A family history of CRC in first-degree relatives was based on self-reporting, as described elsewhere [29]. The questionnaire also covered the regularity of physical exercise: subjects were asked to report the frequency and intensity of recreational exercise, with average daily exercise hours in any intensity calculated and categorized into the three levels of none, and < 0.5 and ≥ 0.5 hours/day.

### Statistical analysis

Odds ratios (ORs) and 95% confidence intervals (CIs) for assessment of the impact of each 8q24 locus, included in the model as an ordinal score (1 to 3), were calculated using multivariable conditional logistic regression models. We explored two models: model 1 was a crude model; model 2 included age and sex plus potential confounders as indicator variables. Confounders considered in model 2 were smoking status (never, former, current moderate, and heavy), drinking habit (non, low, moderate, and heavy), folate consumption by tertile (T1-3), BMI (< 22.5, 22.5 - 24.9, 25.0-27.4 and ≥ 27.5 kg/m^2^), family history of colorectal cancer (yes or no), and regular exercise (none, < 0.5 hour/day, and ≥ 0.5 hour/day). Interactions between rs6983267 assuming linear effect of allele and potential confounders similarly assuming linear effect were assessed in multivariable unconditional logistic regression models to avoid the dropping of subjects in conditional logistic regression models. To assess possible discrepancies between expected and observed haplotypes, accordance with the Hardy-Weinberg equilibrium (HWE) was checked for controls with the χ^2 ^test. Statistical analyses were performed using STATA version 10 (Stata, College Station, TX), with *P*-values < 0.05 considered statistically significant.

## Results

Table 1 shows baseline characteristics of the 481 CRC cases, with an average age of 60 years, and the 962 controls matched for sex and age. Males accounted for 62.4% of subjects. Apart from a family history of CRC in a first-degree relative, potential confounders showed no clear difference between cases and controls. A family history of CRC was significantly more frequent among CRC cases.

**Table 1:** Characteristics of cases and controls

Variables	Cases		Controls		p-values
**Total**	481		962		
**Sex**					1.00
Male	300	62.4%	600	62.4%	
Female	181	37.6%	362	37.6%	
Age (years)					0.803
< 40	20	4.2%	39	4.1%	
40-49	50	10.4%	105	10.9%	
50-59	169	35.1%	328	34.1%	
60-69	164	34.1%	353	36.7%	
70-	78	16.2%	137	14.2%	
Mean age (SD)	60 (10.2)		60 (9.86)		
Site of Cancer					
Colon	245	50.9%			
Rectum	236	49.1%			
Smoking					0.102
None	215	44.7%	493	51.3%	
Low-moderate (< 20 pack-years)	59	12.3%	116	12.1%	
Heavy (≥ 20 pack-years)	203	42.2%	345	35.9%	
Unknown	4	0.8%	8	0.8%	
Drinking					0.695
None	190	39.5%	383	39.8%	
Low (< 5 g ethanl/day)	64	13.3%	125	13.0%	
Moderate (5≤ and < 23 g ethanol/day)	85	17.7%	196	20.4%	
High (≥ 23 g ethanol/day)	135	28.1%	243	25.3%	
Unknown	7	1.5%	15	1.6%	
Daily folate consumption					0.857
T1 (≤ 262.0 μg/day)	153	31.8%	286	29.7%	
T2 (≤ 346.6 μg/day)	158	32.9%	318	33.1%	
T3 (> 346.6 μg/day)	162	33.7%	341	35.5%	
Unknown	8	1.7%	17	1.8%	
Body-Mass Index (BMI) kg/m^2^					0.923
< 22.5	206	42.8%	397	41.3%	
22.5 ≤ and < 25	157	32.6%	314	32.6%	
25 ≤ and < 27.5	73	15.2%	162	16.8%	
≥ 27.5	41	8.5%	79	8.2%	
Unknown	4	0.8%	10	1.0%	
Family history of colorectal cancer in the first degree relatives					0.014
No	453	94.2%	932	96.9%	
Yes	28	5.8%	30	3.1%	
Average recreational exercise					0.329
None	192	39.9%	349	36.3%	
< 0.5 hour/day	194	40.3%	398	41.4%	
0.5 ≤ hour/day	95	19.8%	215	22.4%	

Genotype distributions for 8q24 rs6983267 and rs10090154 are shown in Table 2. Among controls, both genotypes were accordant with the HWE. The minor allele frequency for rs6983267 was 0.338 (G-allele). The age- and sex-adjusted in the allelic model showed an OR of 1.22 (1.04-1.44, p = 0.0144) and the confounder-adjusted model an OR of 1.25 (1.06-1.48, p = 0.0071). Genotypic model showed a significant association only with rs6983267 GG genotype (OR = 1.64, 1.15-2.35, p = 0.0063). In contrast, rs10090154 showed no association with CRC risk. Table 3 shows stratified analyses conducted to explore possible interactions between potential confounders although point estimates for ORs were not static; no significant interactions were seen between the factors examined and rs6983267. The lack of association in those with a positive family history was of interest vis a vis the significant association in those without it, albeit that the number of subjects with a family history was limited.

**Table 2:** Genotypes distribution of 8q24 polymorphisms and odds ratios for the minor alleles and genotypes.

					Allelic model						Genotype model					
						
					Model 1 *1			Model 2 *2			Heterozygote			Minor homozygote		
								
8q24 locus		Genotype			OR	95% CI	p-value	OR^a^	95% CI	p-value	OR^a^	95% CI	p-value	OR^a^	95% CI	p-value
rs6983267 (Minor allele: G, MAF*1 in controls = 0.338)																
	TT	TG	GG	UK*4												
case/control	181/418	222/436	73/107	3/1	1.22	1.04-1.44	0.0144	1.25	1.06-1.48	0.0071	1.19	0.93-1.52	0.1665	1.64	1.15-2.35	0.0063
																
**rs10090154 (Minor allele: T, MAF in controls = 0.153)**																
	CC	CT	TT	UK												
case/control	355/689	112/247	11/23	3/3	0.90	0.72-1.12	0.3443	0.87	0.69-1.09	0.2140	0.83	0.63-1.08	0.1690	0.90	0.43-1.89	0.7854

*1 Crude conditional logistic regression model.

*2 Adjusted for age as continuous variable, drinking (non, low, moderate, heavy, and unknown), smoking (non, moderate, heavy, unknwon), BMI (< 22.5, < 25, < 27.5, 27.5-, unknown), folate in tertile (T1, T2, T3, and unknown), total energy intake, family history of colorectal cancer, average recreational exercise (none, < 0.5 hour/day, 0.5-hour/day) in conditional logisitic regression.

*3 MAF indicates minor allele frequency.

*4 UK indicates the subjects whose genotyping was unsuccessful.

**Table 3:** Stratified analysis according to potential confounding factors for 8q24 rs6983267 genotype

			rs6983267 allelic model						
				
			Model 1*1			Model*2			
					
Exposure	Number of controls with each genotype (TT/TG/GG)	Number of cases with each genotype (TT/TG/GG)	OR*1	95% CI	p-value	OR	95% CI	p-value	Interaction P
**Sex**									0.181
Male	**122/133/43**	**259/270/70**	1.11	0.91-1.37	0.295	1.14	0.93-1.41	0.212	
Female	**61/89/30**	**159/166/37**	1.44	1.10-1.88	0.007	1.34	1.01-1.78	0.040	
**Smoking**									0.401
None	**73/106/35**	**206/232/55**	1.34	1.05-1.70	0.018	1.29	1.01-1.65	0.042	
Low-moderate (< 20 pack-years)	**19/31/9**	**52/52/12**	1.51	0.93-2.43	0.094	1.49	0.89-2.49	0.130	
Heavy (≥ 20 pack-years)	**88/84/29**	**158/147/39**	1.11	0.86-1.43	0.423	1.12	0.86-1.44	0.407	
**Drinking**									0.437
None	**69/92/30**	**166/179/38**	1.36	1.04-1.76	0.023	1.35	1.03-1.77	0.028	
Low (< 5 g ethanl/day)	**23/29/12**	**51/58/16**	1.25	0.81-1.93	0.308	1.44	0.91-2.28	0.124	
Moderate (5≤ and < 23 g ethanol/day)	**34/41/9**	**85/83/27**	0.98	0.67-1.43	0.918	0.99	0.67-1.46	0.941	
High (≥ 23 g ethanol/day)	**55/59/19**	**112/108/23**	1.22	0.89-1.67	0.217	1.22	0.88-1.69	0.231	
**Daily folate consumption**									0.694
T1 (≤ 262.0 μg/day)	**54/73/25**	**112/137/37**	1.14	0.85-1.53	0.375	1.22	0.90-1.66	0.197	
T2 (≤ 346.6 μg/day)	**59/75/22**	**104/141/36**	1.21	0.91-1.61	0.191	1.25	0.93-1.67	0.141	
T3 (> 346.6 μg/day)	**66/72/24**	**157/152/32**	1.24	0.94-1.64	0.135	1.30	0.97-1.73	0.080	
**Body-Mass Index (BMI) kg/m^2^**									0.678
< 22.5	**73/100/32**	**177/176/44**	1.34	1.05-1.72	0.020	1.43	1.10-1.85	0.007	
22.5 ≤ and < 25	**69/69/19**	**128/148/37**	0.93	0.70-1.25	0.663	0.93	0.69-1.25	0.637	
25 ≤ and < 27.5	**23/34/16**	**74/69/19**	1.58	1.06-2.36	0.024	1.67	1.09-2.55	0.018	
≥ 27.5	**18/17/5**	**31/41/7**	0.98	0.54-1.79	0.944	1.06	0.54-2.10	0.863	
Family history of colorectal cancer in the first degree relatives									0.765
No	**169/212/69**	**404/422/105**	1.24	1.05-1.46	0.011	1.26	1.06-1.48	0.008	
Yes	**14/10/4**	**14/14/2**	1.09	0.50-2.38	0.833	0.84	0.29-2.43	0.741	
Average recreational exercise									0.109
None	**77/90/24**	**161/140/48**	1.10	0.85-1.42	0.462	1.13	0.87-1.47	0.356	
< 0.5 hour/day	**73/89/31**	**158/199/40**	1.20	0.93-1.56	0.163	1.20	0.92-1.57	0.174	
0.5 ≤ hour/day	**33/43/18**	**99/97/19**	1.59	1.11-2.28	0.012	1.70	1.15-2.50	0.008	

*1 Odds ratios were adjusted for age and sex in unconditional logistic regression models. Conditional logistic models were not applied because keeping matching in stratficiation gave unstabel estimation.

*2 Odds ratio adjusted for age, sex and all variables in this examination except variable used for stratification.

*3 Interaction term between rs6983267 genotype in score and stratifiying factor in socre was added in model 2.

*4 Subjects were exlucded from analysis because of lack of information, smoking (4 cases and 8 controls), drinking (7 cases and 15 controls), folate (8 cases and 17 controls), and BMI (3 cases and 10 controls)

## Discussion

In this study, we found that the G allele in rs6983267 was associated with a significantly increased risk of CRC in a Japanese population. This finding is consistent with those from previous GWASs [6,9,11] and a pooled analysis [12], as reviewed in Table 4, which reported the consistency of this association with CRC and colorectal adenoma in populations with European ancestry. The only previous study of rs6983267 in a population with Asian ethnicity (Japanese-American) was that by Haiman et al [6], and to our knowledge the present study is the first indication in Japanese living in Japan. Tenesa et al. reported significant association with rs7014346 in 8q24, which is in high linkage disequilibrium with rs6983267, in Japanese population [19], supporting significant association between the rs6983267 in CRC in Japanese. Recent advances in genetic analysis have enabled a comprehensive approach to identifying disease susceptibility loci. The consistency of findings in this and the previous studies warrants the usefulness of the GWAS approach across ethnicities. We also evaluated potential interactions between common background factors and rs6983267, but found no significant interaction between them. Berndt et al. also reported a lack of interaction between rs6983267 and age, sex, smoking, family history of CRC and cancer site [12]. The consistency of this finding indicates that rs6983267 is associated with CRC risk independently of common risk factors.

**Table 4:** Review of results of 8q24 rs6983267 for colorectal cancer in allelic model.

Author	Year	Case/Control	Country	Study subjects	Per allele OR (95%CI)	Adjustment
**Haiman et al.**	2007	**1,807/5,511**	**USA**	**Pooled**	**1.25 (1.12-1.38)**	Sex
		217/1,049		African American	1.37 (0.98-1.91)	Sex
		381/1,197		Japanese American	1.13 (0.96-1.34)	Sex
		61/347		Native Hawaiian	1.59 (1.02-2.47)	Sex
		251/1,007		Latinos	1.26 (1.02-1.55)	Sex
		214/973		European Americans	1.28 (1.03-1.58)	Sex
**Tomlinson et al.**	2007	**7,954/6,206**	**UK**	**CRC pooled**	**1.21 (1.15-1.27)**	Crude
		620/960		Panel A CRC White UK residents	1.38 (1.19-1.59)	Crude
		4,361/3,752		Panel B White UK residents	1.19 (1.12-1.26)	Crude
		1,901/1,079		Panel C	1.21 (1.09-1.35)	Crude
		1,072/415		Panel D European Ancestry	1.13 (0.96-1.33)	Crude
		**1,425/2,255**		**Adenoma pooled**	**1.22 (1.10-1.34)**	Crude
		407/1,027		Panel A Adenoma White UK residents	1.53 (1.29-1.81)	Crude
		607/765		Panel E	1.05 (0.90-1.23)	Crude
		411/463		Panel F	1.13 (0.93-1.37)	Crude
**Poynter et al.**	2007	**1,339/2,191**	**USA**	**Population -- based**	1.11 (0.96-1.29)	Age and sex
**Tuupanen et al.**	2008	996/1,012	**Finland**	**Population-based**	1.22 (1.08-1.38)	Crude
**Berndt et al.**	2008	**3,134/4,454**	**USA**	**Colorectal neoplasms pooled**	**1.16 (1.07-1.25)**	Age, sex, and study
		547/1,656		PLCO	1.17 (1.01-1.35)	Age and sex
		1,174/1,293		PLCO adenoma	1.24 (1.11-1.39)	Age and sex
		364/363		PLCO II	0.93 (0.75-1.16)	Age and sex
		544/542		NHS	1.10 (0.93-1.30)	Age and sex
		505/600		Minnesota	1.21 (1.01-1.44)	Age and sex
**Ghousaini et al.**	2008	**2,299/2,284**	**UK**	**Cases from prospective study at East Anglia**	**1.27 (1.16-1.37)**	Crude
**Lie et al.**	2008	**561/721**	USA	Colon cancer cases from SEER Kentucky	**1.69 (1.19-2.40)**	Age, sex, BMI, and NSAID use.
				Cuacasian	1.61 (1.36-2.30)	Age, sex, BMI, and NSAID use.
**Schafmayer**	2009	**2,713/2,718**	Germany	Colorectal cancer cases with German ancestry	**1.22 (1.13-1.31)**	Crude
**Curtin et al.**	2009	**1,069/1,040**	**USA/UK**	**Colorectal cancer cases from USA/UK**	**1.17 (1.03-1.32)**	Crude
**Our study**		**481/962**	**Japan**	**HERPACC II participatns (Japanese)**	**1.22 (1.04-1.44)**	Age and sex
					1.26 (1.06-1.48)	Age, sex, drinking, smoking, BMI, folate consumption, energy, physical exercise, and family history of CRC

Rs6983267 was originally identified using a non-hypothesis-based approach, and evidence has suggested a possible biological mechanism behind this observed association. The rs6983267 polymorphism resides 15 kb upstream of a processed pseudogene (*POU5F1P1*) of the POU-domain factor gene, *POU5F1*, which encodes transcription factor OCT4, with 97.5% shared identity [30]. OCT4, a transcript of *POU5F1*, plays a role in maintaining stem cell pluripotency, self-renewal and chromatin structure in stem cells [31], and promotes tumor growth in a dose-dependent manner [32]. A conserved POU5F1-binding site I at the 5' promoter region of the WNT-signaling gene, *FZD5*, has been reported [33]. Tomlinson et al. reported the expression of either POU5F1 or POU5F1P1 in cell lines and primary CRCs [9], while Suo et al. similarly reported the expression of these genes in cancer cell lines and cancer tissues [30]. Given that OCT4 pseudogenes in mice are reported to mediate stem cell regulatory function [34], it is possible to hypothesize that OCT4 pseudogenes, including *POU5F1P1*, might play a role in stem cell proliferation. However, no difference in expression according to rs6983267 status was observed [9]. Berndt discussed the potential contribution of *MYC*, which is located > 300 KB distant to rs6983267[12]. Recently, Pomerantz et al. reported rs6983267 displays a difference in binding of transcription factor 7-like 2 (TCF7L2) leading to a different physical interaction with *MYC *[35]; however, Tuupanen et al. failed to find clear association between rs6983267 genotype and *MYC *expression. There still remains controversy between *MYC *and rs6983267 requiring further studies. Moreover, Tuupanen et al. reported rs6983267 affects a binding site for the Wnt-regulated transcription factor (TCF4), with the risk allele G showing stronger binding *in vivo *and *in vitro*. Overall, these findings indicate that the possible biological mechanism behind the effect of rs6983267 polymorphism on CRC carcinogenesis requires further study.

We did not observe any association with rs10090154 (OR = 0.90) on the contrary to the results from Multi-ethnic cohort study [6]. The point estimate for minor allele in the previous study was 1.41 (95%CI: 1.14-1.75). Following case-control study for Japanese American in Hawaii showed lack of association (OR = 1.07, 95%CI: 0.78-1.48)[6]. Inconsistency across studies might come from the finding in the original GWAS was by chance although threshold in statistical significance was high enough. Or, statistical power in following studies including ours was not good enough. By all means, more evidence is needed to clarify significance of the locus.

Several potential limitations of the present study require consideration. First, use of hospital-based control in this study for potential cause of selection bias. We used non-cancer patients at our hospital as controls, given the likelihood that our cases arose within this population base. Moreover, we previously showed that individuals selected randomly from our control population were similar to the general population in terms of baseline characteristics [22]. Given the similarity in minor allele frequency between our controls and that in the HapMap database for Japanese, it is reasonable to assume the external validity of our study results to the general population. Second, as with other case-control studies, this study may have suffered from information bias: although the questionnaires were completed before the diagnosis in our hospital, some patients referred from other institutions might have known their diagnosis. Lack of interaction needs careful interpretation because confounders assessed in this study showed no association with CRC risk by themselves.

## Conclusion

Our present investigation showed that rs6983267 in 8q24 is an independent risk factor of CRC in a Japanese population. Further studies to clarify the biological mechanisms of this association are warranted.

## Competing interests

The authors declare that they have no competing interests.

## Authors' contributions

MW carried out the molecular genetic studies. JY carried out the immunoassays. MT participated in the sequence alignment. TS, TK, HT, and KT participated in the design of the study and helped to draft the manuscript. KS, KK, YK, TH and YY participated in the enrollment and conduct of the study. KM conceived of the study, participated in its design and statistical analyses. All authors read and approved the final manuscript.

## Pre-publication history

The pre-publication history for this paper can be accessed here:

http://www.biomedcentral.com/1471-2407/9/379/prepub
